# Deconstructing Pancreatic Cancer Using Next Generation-Omic Technologies–From Discovery to Knowledge-Guided Platforms for Better Patient Management

**DOI:** 10.3389/fcell.2021.795735

**Published:** 2022-01-13

**Authors:** Daniel Schreyer, John P. Neoptolemos, Simon T. Barry, Peter Bailey

**Affiliations:** ^1^ Institute of Cancer Sciences, University of Glasgow, Scotland, United Kingdom; ^2^ Department of General, Visceral, and Transplantation Surgery, Heidelberg University Hospital, Heidelberg, Germany; ^3^ Bioscience, Oncology R&D, AstraZeneca, Cambridge, United Kingdom; ^4^ Section Surgical Research, University Clinic Heidelberg, Heidelberg, Germany

**Keywords:** precision medicine, omic, pancreatic cancer, molecular profiling and subtyping, next-generation sequencing, single-cell ‘omics, spatial transcriptomics, cancer biomarker

## Abstract

Comprehensive molecular landscaping studies reveal a potentially brighter future for pancreatic ductal adenocarcinoma (PDAC) patients. Blood-borne biomarkers obtained from minimally invasive “liquid biopsies” are now being trialled for early disease detection and to track responses to therapy. Integrated genomic and transcriptomic studies using resectable tumour material have defined intrinsic patient subtypes and actionable genomic segments that promise a shift towards genome-guided patient management. Multimodal mapping of PDAC using spatially resolved single cell transcriptomics and imaging techniques has identified new potentially therapeutically actionable cellular targets and is providing new insights into PDAC tumour heterogeneity. Despite these rapid advances, defining biomarkers for patient selection remain limited. This review examines the current PDAC cancer biomarker ecosystem (identified in tumour and blood) and explores how advances in single cell sequencing and spatially resolved imaging modalities are being used to uncover new targets for therapeutic intervention and are transforming our understanding of this difficult to treat disease.

## Introduction

Large scale genomic sequencing of pancreatic ductal adenocarcinoma (PDAC) has revealed subgroups of patients that share clinically and biologically relevant molecular similarities (Jones et al., 2008; Biankin et al., 2012; Waddell et al., 2015; Bailey et al., 2016; Raphael et al., 2017; ICGC/TCGA Pan-Cancer Analysis of Whole Genomes Consortium 2020). An estimated 30–40% of PDAC tumours harbour an “actionable mutation” that can be matched to a known therapeutic regimen (Chantrill et al., 2015; Lowery et al., 2017; Aung et al., 2018; Singhi et al., 2019; Pishvaian et al., 2020). Precision oncology trials are now testing whether single biomarker drug combinations can be delivered in clinically relevant timeframes (Pishvaian et al., 2020). Beyond actionable mutations, “bulk” transcriptomic profiling has identified patient tumour subtypes with distinct biology that are associated with beneficial responses to standardised adjuvant chemotherapy (Collisson et al., 2011; Moffitt et al., 2015; Bailey et al., 2016; Raphael et al., 2017; Tiriac et al., 2018; O’Kane et al., 2019; Chan-Seng-Yue et al., 2020). Furthermore, transcriptomic signatures that are predictive of patient responses to standardised chemotherapy, e.g., gemcitabine, are now being tested in clinical trials to improve patient outcomes by identifying the optimal patient-specific chemotherapy.

Next generation single cell and single nucleus sequencing methods are augmenting “bulk” sequencing data to provide a comprehensive map of tumour cell communities and driver mutation dynamics. These methods are being deployed to investigate chemotherapy resistance and metastasis. In addition, advances in spatially resolved transcriptomics and multiplexed imaging modalities are providing fundamental information about neoplastic-immune cell interactions, with the potential to deliver precision immuno-oncology for PDAC.

Despite advances in surgical techniques and the optimisation of chemotherapy regimens, the overall 5-year survival for PDAC is only 10% (Siegel et al., 2021). PDAC is commonly diagnosed at advanced stages, at which time surgical resection is not an option (Mizrahi et al., 2020). As a result, there is considerable interest in the development of advanced genomic methodologies for the early detection of PDAC. The detection of somatic mutations and methylation signatures in circulating tumour DNA (ctDNA), which can be isolated from patient blood, provides an important first step in the development of minimally invasive blood tests for early PDAC diagnosis. In addition, the advancement of methods for the detection and characterisation of other blood-borne analytes will enable real-time monitoring of therapy response without the need for invasive biopsies.

**FIGURE 1:**
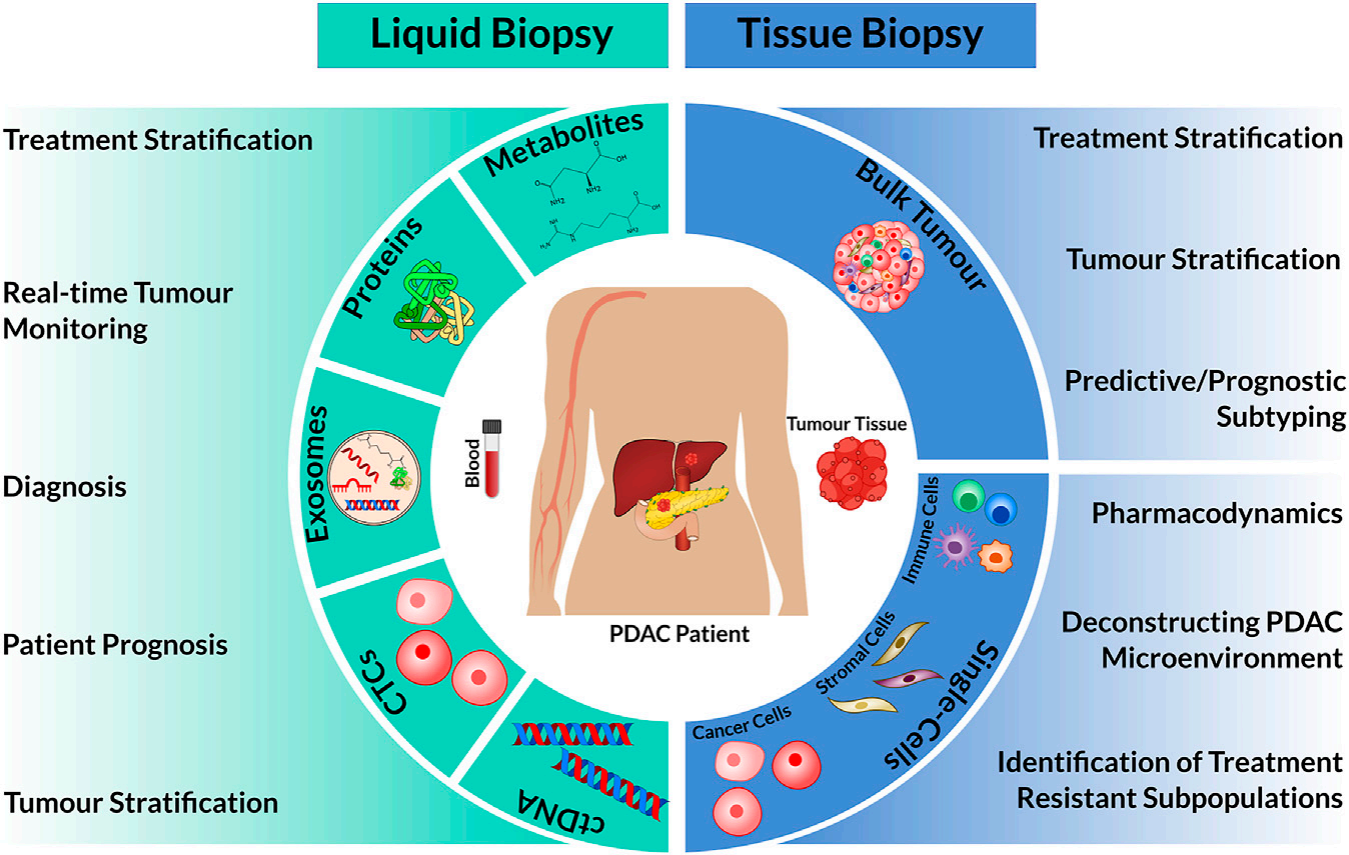
Overview of patient materials isolated from liquid and tissue biopsies for omic analysis and their clinical utility. The circulating biomarkers: cell-free tumour DNA (ctDNA), circulating tumour cells (CTCs), exosomes, proteins or metabolites can be isolated and characterized from blood specimens. In comparison, tissue biopsies extract bulk tumour tissue, which can be used as a whole (Bulk Tumour) or dissociated into single cells. In combination with omic technologies, these biological materials can have diverse clinical use **(left and right)**.

The development and implementations of precision oncology for PDAC requires the appropriate deployment of cancer biomarkers into routine clinical care. Cancer biomarkers detected in tissue biopsies and/or blood or other fluids can help to diagnose early PDAC or its recurrence, be prognostic or predict a patient’s response to specific drugs (Figure 1). Unfortunately, patient management for PDAC is currently hampered by a lack of defining biomarkers. This review examines the current PDAC cancer biomarker ecosystem and describes new “omic” technologies that promise to uncover new prognostic biomarkers by deconstructing patient tumours at single cell resolution.

## Genomic Profiling Identifies Driver Events and Actionable Mutations in PDAC

Large scale genomic sequencing studies have transformed our understanding of the genomic events that shape PDAC (Jones et al., 2008; Biankin et al., 2012; Waddell et al., 2015; Bailey et al., 2016; Raphael et al., 2017; Connor et al., 2019; ICGC/TCGA Pan-Cancer Analysis of Whole Genomes Consortium 2020). The PDAC mutational driver landscape is dominated by recurrent, predominantly overlapping mutations in KRAS, TP53, SMAD4, and CDKN2A (>50%) with a subset of additional genes including KDM6A, MLL3, ARID1A, TGFBR2, RBM10, and BCORL1 recurrently mutated in 5–10% of patient samples (Waddell et al., 2015; Bailey et al., 2016; Collisson et al., 2019). Most single gene biomarkers that can be matched to a drug such as ERBB2 amplification, BRAF gene fusions/mutations and mutations in DNA damage repair (DDR) genes BRCA1, BRCA2, or PALB2 are found in less than 4% of patients (Harada et al., 2008; Jones et al., 2008; Biankin et al., 2012; Witkiewicz et al., 2015).

Aggregation of genomic aberrations into common pathways and/or based on therapeutic intervention has helped to identify several “actionable molecular phenotypes” that are currently under clinical investigation (Collisson et al., 2019). A meta-analysis of 21,842 PDAC genomes has estimated that the pooled prevalence of germline and somatic mutations in DNA damage response genes (i.e., BRCA1, BRCA2, PALB2, ATM, ATR, CHEK2, RAD51, and FANC) that cause homologous recombination deficiency (HRD) is between 14.5 and 16.5% (Casolino et al., 2021). Recent evidence also suggests that surrogate biomarkers of HRD, such as unstable genomes (determined by SV analysis) and BRCA mutational signatures (BRCAness phenotype) may identify even greater numbers of HRD patients (24–44%) (Waddell et al., 2015; Casolino et al., 2021). HRD is a putative biomarker of therapeutic response to DNA damaging agents such as platinum and PARP inhibitors suggesting that a large segment of the PDAC patient population may benefit from these therapies (Collisson et al., 2019; Casolino et al., 2021).

Germline testing for the identification of known hereditary cancer syndromes, e.g., BRCA1/2 is increasingly recommended for patients with PDAC (Margaret A. Tempero et al., 2021). FOLFIRINOX (5-Fluorouracil, Irinotecan, and Oxaliplatin) has been shown to elicit significant responses in advanced PDAC patients who harbour germline BRCA1 and BCRA2 mutations (Golan et al., 2014; Wattenberg et al., 2020). Olaparib is FDA-approved for maintenance therapy in germline BRCA1/2 PDAC patients after platinum-based chemotherapy with response or stable disease (Golan et al., 2014). Maintenance trials with other PARP inhibitors are ongoing, with preliminary data showing improved responses in patients with advanced platinum sensitive BRCA or PALB2 mutated PDAC after treatment with Rucaparib (Rubraca) as a maintenance monotherapy (NCT 03140670).

Therapeutically targeting HRD may extend beyond germline carriers to patient groups harbouring somatic mutations in DNA damage repair genes or that exhibit a “BRCAness phenotype” (Casolino et al., 2021). Phase II precision oncology trials are now assessing whether single agent Olaparib therapy is effective in advanced PDAC patients that do not harbour germline BRCA1/2 mutations (NCT02677038). In addition, several early phase clinical trials are testing whether PARP inhibitors in combination with either standard chemotherapy (NCT03337087; NCT04228601) or other targeted therapies (NCT03842228; NCT04005690; NCT03682289) are effective in treating germline and/or somatic HRD in advanced PDAC (Singh et al., 2021).

Several alternative therapeutic strategies targeting HRD PDAC are also currently being explored. Patient-derived cell lines (PDCLs) harbouring germline and/or somatic mutations in BRCA1, BRCA2, PALB2 are sensitive to cell cycle checkpoint inhibitors specific for CHECK1, WEE1, ATR, and PLK4 (Dreyer et al., 2021). Synergies between PARP and ATR or DNA PKcs inhibitors can lead to synthetic lethality in ATM-deficient Genetically Engineered Mouse Models (GEMMs) and PDAC cell lines (Gout et al., 2021). Lessons from ovarian cancer also suggest that concurrent treatment of cell lines with PARP inhibitors followed by cell cycle checkpoint inhibitors can induce DNA damage and replication stress in both HRD and non-HRD tumour cells (Fang et al., 2019). High replication stress is commonly associated with greater sensitivity to cell cycle checkpoint inhibitors (Fang et al., 2019; Dreyer et al., 2021). Recent evidence in PDAC PDCLs demonstrates that cells having a Basal-like transcriptomic subtype (see below) may exhibit higher endogenous replication stress than those having a Classical subtype (Dreyer et al., 2021). Importantly, Basal-like PDCLs were found to exhibit greater sensitivity to cell cycle checkpoint inhibitors in a non-HRD dependent manner. These studies suggest that sequential treatment of PDAC with DNA damaging agents, either via standardised chemotherapy or PARP inhibitors, followed by cell cycle checkpoint inhibitors may be an effective treatment strategy for PDAC. In addition, stratification of patients by transcriptomic subtype may augment treatments that exploit replication stress.

## Transcriptomics–Identification of Prognostic and Predictive Omic Subtypes Using Bulk and LCM-Derived Patient Material

The recent identification of intrinsic transcriptomic subtypes of PDAC provides an alternative strategy for patient treatment selection–reviewed in depth by Xu Z et al. (2021). Transcriptomic profiling has identified 2 broad intrinsic PDAC classes, namely Classical and Basal-like with Basal-like tumours associated with significantly poorer outcomes and late-stage disease (Moffitt et al., 2015; Collisson et al., 2019). These classes are delineated by the differential expression of pancreatic specific transcription factors, such as GATA6, PDX1, and HNF1A, that act to specify and maintain Classical pancreatic identify and which are lost in Basal-like tumours (Collisson et al., 2011; Moffitt et al., 2015; Bailey et al., 2016; Raphael et al., 2017).

Findings from both COMPASS trial and pre-clinical models of PDAC demonstrate that Basal-like tumours are less likely to respond to standard-of-care chemotherapy than Classical tumours (Tiriac et al., 2018; Chan-Seng-Yue et al., 2020; Rashid et al., 2020). Transcriptional profiling of pancreatic cancer patient-derived organoids has identified treatment stratification signatures (TSS) that can identify best responders to gemcitabine-based therapy, or mFOLFIRINOX (Tiriac et al., 2018). When retrospectively applied to transcriptomic data from laser capture micro-dissected tumour tissues, obtained from either resected or advanced cases, these TSS were able to predict improved responses to mFOLFIRINOX or gemcitabine/nAb-Paclitaxel. Basal-like tumours were found to be significantly enriched in the mFOLFIRINOX non-sensitive group (Tiriac et al., 2018). As a corollary, it has recently been shown that GATA6 expression in tumours from patients with advanced disease (using RNA *in situ* hybridization) can discriminate Classical and Basal-like tumours. Importantly, the best progression-free survival in patients with advanced disease was found in GATA6-positive Classical tumours given mFOLFIRINOX (O’Kane et al., 2020).

Detailed integrative analysis (combing both transcriptomic and genomic analyses) has demonstrated that while transcriptomic profiling can stratify PDAC into Classical and Basal-like subtypes the genomes of tumours falling within these same transcriptomic subtypes may exhibit considerable intra-tumoral heterogeneity (ITH) (Chan-Seng-Yue et al., 2020). Recent evidence demonstrates that both the Classical and Basal-like subtypes comprise at least 2 distinct subclusters that are driven by specific copy number (CN) gains in genes such as mutant KRAS and GATA6. Minor and major KRAS CN imbalances can further stratify the Basal-like subtype into 2 distinct subclusters, with minor KRAS CN imbalances associated with Primary disease (Stages I–III) and major KRAS CN imbalances linked to metastasis (Stage IV) and Squamous-like gene expression programmes. Importantly, Stage IV tumours with Major mutant KRAS CN gains were found to be significantly more resistant to FOLFIRINOX compared to those with minor mutant KRAS CN gains (Chan-Seng-Yue et al., 2020). These findings are incredibly informative in that they support a model of PDAC in which ongoing genomic instability during disease progression gives rise to different molecular phenotypes.

These findings also point to the potential utility of GATA6 and KRAS CN gains as biomarkers of disease progression, recurrence and/or opportunity for selective therapy (Miyabayashi et al., 2020).

## Epigenomic Profiling for the Identification of Therapeutic Opportunities and Patient Prognosis

The 2 major transcriptomic subtypes of PDAC are defined by distinct epigenetic states. Bailey et al. were the first to demonstrate that distinct DNA methylation patterns define the Squamous and Classical subtypes. Squamous subtype tumours were found to exhibit a pattern of CpG DNA methylation significantly correlated with the downregulation of pancreatic specific transcription factors that control pancreatic cell fate determination (PDX1, GATA6, and HNF1A), and the activation of multigene programmes controlled by MYC and ΔNTP63 that drive Squamous-like differentiation (Bailey et al., 2016).

Lomberk et al., generated comprehensive chromatin state maps to characterise the epigenetic landscape of patient-derived PDAC tumour xenograft models (Lomberk et al., 2018). This analysis demonstrated that critical regulatory hubs, so called “super-enhancers”, exhibit subtype specific activity. Mapping of super-enhancers to genes in the Classical subtype demonstrated that active super-enhancers regulate the expression of pancreatic specific TFs such as GATA6, HNF4A, FOS, FOXP1, FOXP4, KLF4, ELF3, and CUX1. In contrast, Basal-like specific super-enhancer regulation was associated with the hepatocyte growth factor receptor MET, MYC, and ΔNTP63. Interestingly, MET inhibition in Basal-like tumours resulted in a switch from Basal-like to Classical-like gene expression programmes. Subsequent mechanistic studies demonstrated that ΔNTP63 binds to the distal enhancer elements of genes important for Squamous-like differentiation (Somerville et al., 2018). Over-expression of ΔNTP63 in Classical cell lines was sufficient to reprogramme Classical cells towards a Squamous-like state. Together these studies implicate super-enhancers and the TFs that they regulate as critical determinants in PDAC subtype-specific gene expression. This data also suggests that phenotypic heterogeneity may be regulated by epigenetic elements and that these elements may be modulated by therapy (Lomberk et al., 2018). Therapies designed to modify the PDAC epigenome may, therefore, expose a therapeutic vulnerability for subsequent targeted intervention. Alternatively, therapies may reprogramme the epigenome and induce aggressive phenotypes or contribute to therapy resistance. Whether therapy induced epigenetic changes are hard wired or can be rewired to generate an original phenotype remains to be determined.

Mutations in chromatin modifiers, such as KDM6A, ARID1A, ARID1B, PBRM1, SMARCA2, PBRM1, SMARCA2, SMARCA4, or MLL2 are a feature of PDAC (Waddell et al., 2015). Mutations in KDM6A are enriched in the Squamous transcriptomic subtype, underpinning the importance of chromatin modifiers in shaping PDAC epigenetic states (Bailey et al., 2016). A Genetically Engineered Mouse Model of PDAC demonstrates that genetic inactivation of KDM6A rewires the epigenetic landscape of PDAC causing the aberrant activation of super-enhancers regulating the expression of ΔNTP63, MYC, and RUNX3. Activation of these TFs may subvert pancreatic identity and induce Squamous-like differentiation phenotypes (Andricovich et al., 2018). This work also demonstrates that KDM6A null tumours can be selectively targeted by the (Bromodomain and Extra Terminal) BET inhibitor JQ1. The actionability of mutations in other chromatin modifiers remains an open question. In other cancer types, cells harbouring mutations in ARID1A have been shown to exhibit defective DNA damage repair and increased sensitivity to ATR and PARP inhibitors (Williamson et al., 2016). Furthermore, ARID1A mutations have been demonstrated to induce immune phenotypes susceptible to immune checkpoint inhibitors in orthotopic and intraperitoneal ovarian cancer mouse models (Shen et al., 2018). Mutations in chromatin modifiers are found in up to 10% of all PDAC and represent a large yet underexplored therapeutic opportunity for PDAC.

Recent evidence also implicates the 5-methylcytosine hydroxylase TET2 as a critical modulator of PDAC epigenetic states (Eyres et al., 2021). Genome-wide epigenetic mapping of 5-hydroxymethylcytosine (5 hmc) DNA modifications in resected PDAC samples demonstrated that Squamous-like PDAC is associated with loss of 5 hmc at pancreatic specific gene loci, including GATA6. Loss of 5 hmc was directly associated with the concomitant downregulation of TET in Squamous PDAC samples. Moreover, TET2 expression was linked to SMAD4 mutational status, with SMAD4 inactivation associated with loss of 5 hmc and downregulation of GATA6. Stabilising TET2 expression by treatment with metformin and vitamin C was sufficient to restore 5 hmc and GATA6 levels and increase biomarkers of Classical PDAC. These findings further highlight the importance of epigenetic mechanisms in governing PDAC transcriptional phenotypes and the ability of epigenetic states to be modulated by small molecules (Eyres et al., 2021).

Divergent epigenetic states not only discriminate PDAC subtypes, but also underpin tumour progression and metastases (McDonald et al., 2017; Lomberk et al., 2019). Combined genomic and epigenomic analyses using low passage cell lines derived from matched primary and metastatic PDAC demonstrated that cell lines derived from distant metastatic sites exhibit widespread epigenetic reprogramming when compared to cells of the primary tumour. Metastatic cell lines exhibited epigenetic programmes consistent with increased glycolytic dependency via the oxidative branch of the pentose phosphate pathway (oxPPP). Importantly, epigenetic reprogramming was observed in the absence of metastasis-specific driver mutations suggesting that epigenetic changes alone may drive adaption to metastatic niches (McDonald et al., 2017).

Brunton et al. used Assay for transposase Accessible Chromatin with high-throughput sequencing (ATAC-seq) to identify additional PDAC subgroups with potential therapeutic utility. This study demonstrated that differential chromatin accessibility, as assessed by ATAC-seq, can predict responsiveness and tolerance to GSK3β inhibitors in the Squamous subtype of PDAC (Brunton et al., 2020). ATAC-seq has also been applied to resected PDAC samples to identify a chromatin accessibility signature predictive of disease-free survival. Together these studies provide compelling evidence that epigenetic states play an important role in PDAC progression and response to therapy. The application of methodologies, such as ATACseq, and newly developed ATAC-array (Dhara et al., 2021), in different clinical settings, may uncover important new therapeutic opportunities for yet undefined patient subgroups.

## Deconstruction of PDAC at Single Cell Resolution

### Defining Actionable Segments Using scRNAseq and Spatial Transcriptomics

The modelling of PDAC subtypes from bulk tumour RNA profiling has been challenged by a complex admixture of expressed transcripts representing a diversity of normal and neoplastic cell types (Moffitt et al., 2015; Bailey et al., 2016; Collisson et al., 2019). PDAC classification schemes using bulk RNAseq have either attempted to define PDAC subtypes by including all expressed transcripts–and modelling tumour complexity as a whole or by enriching epithelial-derived cancer signals using informed informatic approaches or via laser capture microdissection (LCM) of the cancer-epithelium from abundant cancer stroma (Collisson et al., 2011; Moffitt et al., 2015; Bailey et al., 2016; Raphael et al., 2017; Puleo et al., 2018; Maurer et al., 2019; Chan-Seng-Yue et al., 2020).

The consensus classification of PDAC into 2 broad tumour cell intrinsic transcriptomic subtypes, namely Classical and Basal-like, is largely based on the later approach which favours the enrichment of gene transcripts that are representative of neoplastic gene programmes (Moffitt et al., 2015). Classification schemes that have defined additional subtypes such as aberrantly differentiated endocrine exocrine (ADEX), exocrine-like and/or immunogenic have been contested on the basis that these subtypes represent non-malignant stromal or normal cell contamination (Collisson et al., 2011; Bailey et al., 2016). Despite these criticisms, there is still some debate about the contribution of “normal” exocrine-like tumour tissue to PDAC. Further, the relationship between neoplastic cells and stromal cells remains unclear, although there is now a greater appreciation that diverse signals from epithelial-derived cancer cells shape the PDAC tumour microenvironment (Tape et al., 2016; Candido et al., 2018).

The 2-subtype consensus classification for PDAC, therefore, belies a complex tumour architecture made up of diverse cell types including “normal” adjacent, neoplastic, endothelial, Cancer-Associated Fibroblasts (CAFs) and immune cells. The contribution of these different cell types to PDAC, their inter-relationships and the importance of CAF and immune cells as mediators of standardised chemotherapy remain critical questions. single cell RNAseq (scRNAseq) is helping to address these questions by deconstructing tumours at single cell resolution (Peng et al., 2019; Hwang et al., 2020; N. G.; Steele et al., 2020; Hutton et al., 2021; Raghavan et al., 2021; Chen et al., 2021). Used in combination with spatially resolved transcriptomics and state-of-the-art multiplexed imaging platforms, new insights are emerging about neoplastic phenotypes (subtypes), the interplay between neoplastic and stromal cell populations and the evolution of resident tumour cell populations during disease progression, metastasis and in response to therapy (Raghavan et al., 2021; N. G.; Steele et al., 2020).

### PDAC Subtypes at Single Cell Resolution

scRNAseq analysis of treatment naïve PDAC has demonstrated that Classical and Basal-like neoplastic cell phenotypes co-exist in the same tumour. Single cell profiling of biopsied liver metastatic lesions and primary PDAC has identified “hybrid” neoplastic cell populations that share common Classical and Basal-like gene expression profiles, corroborating some earlier bulk RNA expression studies (Chan-Seng-Yue et al., 2020; Hwang et al., 2020). Informatic approaches that infer the trajectories of single cell populations have also revealed that Classical and Basal-like neoplastic phenotypes may represent a dynamic disease continuum with the Basal-like state enriched for mesenchymal and stem-like programmes (Hwang et al., 2020; Raghavan et al., 2021). Recent findings have demonstrated that PDAC neoplastic cells may undergo dynamic phenotypic transitions after chemoradiation therapy (CRT) (Hwang et al., 2020). Single nucleus RNAseq analysis of treatment naïve resected and neoadjuvant CRT patient samples demonstrated a shift from a Classical-like cell phenotype towards an “induced” Basal-like phenotype following CRT (Hwang et al., 2020). This finding suggests that therapeutic resistance may involve a transition towards more Basal-like states and is supported by earlier *ex vivo* findings demonstrating Basal-like phenotypic transitions following treatment of PDAC cell lines with FOLFIRINOX (Porter et al., 2019). Interestingly, CRT was also found to selectively enrich for a subset of neoplastic cells that exhibit acinar and neuroendocrine-classical like phenotypes. These cell phenotypes are reminiscent of the ADEX subtype, identified in bulk RNA studies, and are defined by overlapping sets of genes (Bailey et al., 2016; Hwang et al., 2020).

### PDAC TME and Spatially Resolved Targetable Interactions

#### PDAC Immune Targets

The PDAC tumour microenvironment (TME) plays a fundamental role in disease progression and response to therapy (Candido et al., 2018; Sahai et al., 2020; N. G.; Steele et al., 2020; Hutton et al., 2021). The PDAC TME is generally considered “immunologically cold” with a scarcity of CD8^+^ T cells and a highly immunosuppressive microenvironment rendering most tumours recalcitrant to immunotherapy (Neesse et al., 2015; Ho, Jaffee, and Zheng 2020).

The deconvolution of bulk RNA data using validated gene signatures, that define specific immune cell types and/or phenotype, has demonstrated that T cell, B cells, and myeloid cells contribute to complex patient immune profiles. Immunophenotyping of PDAC samples using multimodal single resolution methodologies, such as scRNAseq, spatial transcriptomics and multiplexed immunofluorescence, has revealed that Classical and Basal-like cell phenotypes are associated with distinct immune microenvironments. Principally, Basal-like cells are associated with increased macrophage infiltration and loss of cytotoxic T cell subsets in both primary and metastatic micro-niches (Raghavan et al., 2021). These findings suggest that Basal-like microenvironments may respond to immunotherapies that specifically target tumour-associated macrophages, such as CSF1R inhibitors (Zhu et al., 2014). Genetic context may be important here and the ability of DDR and MSI tumours to drive distinct immune profiles independent of an established neoplastic subtype remain unknown.

Tumour infiltrating T cells are associated with increased overall survival in PDAC and can potentially predict immunotherapy response (Carstens et al., 2017; Lohneis et al., 2017). Single cell analysis has demonstrated that CD8^+^ T cell tumour infiltration is inversely correlated with myeloid cell enrichment (N. G. Steele et al., 2020). This analysis has also revealed that tumour infiltrating CD8^+^ T cells exhibit exhausted phenotypes and that diminished CD8^+^ T cell fitness increases with disease progression. Exhausted CD8^+^ T cell signatures were associated with increased expression of the immune checkpoint TIGIT. The ligand for TIGIT, PVR was expressed in tumour, endocrine, endothelial cells and myeloid subsets, supporting the observation that myeloid cells promote immunosuppression in PDAC. Single cell data also demonstrates that immune checkpoint receptors are heterogeneously expressed between patients (N. G. Steele et al., 2020). In addition, recent studies have established that primary tumour and metastatic lesions support distinct immune infiltrates, which is largely heterogeneous between patients and also shaped by the metastatic niche (C. W. Steele et al., 2016; Lin et al., 2020; Raghavan et al., 2021; Ho et al., 2021). These data highlight the complexity of individual patient immune microenvironments and suggest that therapeutic approaches targeting immune checkpoints may need to be tailored to individual PDAC patients (N. G. Steele et al., 2020). It is also presently unclear how immune microenvironments change during patient treatment. Therefore, longitudinal single cell studies charting fluctuations in immune infiltrates and neoplastic-immune cell interactions will be an important next step in the development of new immunotherapies for PDAC.

Multiple studies have demonstrated that genomic drivers of PDAC, such as KRAS and MYC, can modulate the TME and support highly immunosuppressive microenvironments (Dey et al., 2020; Muthalagu et al., 2020; Ischenko et al., 2021). Biomarkers of immunotherapy response, however, are limited in PDAC. Hypermutated mismatch repair-deficient tumours which make up around 1% of PDAC are likely to respond to immune checkpoint inhibition (Le et al., 2017). While BRCA1 and BRCA2 deficient tumours are associated with increased immune infiltrates, rates of response to ICI are low. Recent evidence in mouse models of breast and colorectal cancer, suggest that BRCA2-deficient tumours are more susceptible to ICIs than BRCA1-deficient tumour (Samstein et al., 2020; Zhou and Li 2021). In addition, loss of CDKN2A, which is a feature of PDAC, has been identified as a biomarker of immune checkpoint therapy resistance in urothelial carcinoma (Nassar et al., 2021). These studies highlight a diversity of genomic events that may drive differential responses to ICIs and suggest that immunotherapy based on a matched genomic biomarker may be complex.

#### PDAC CAF Targets

CAFs are key components of the tumour microenvironment and are emerging as important therapeutic targets. CAFs are implicated in diverse functions such as matrix deposition and remodelling, reciprocal signalling with neoplastic cells and crosstalk with infiltrating T cells (Sahai et al., 2020). Reciprocal signalling between CAFs and PDAC cell lines grown in co-culture has been shown to promote epigenetic changes in PDAC cells and induce transcriptional and metabolic programmes that intersect with oncogenic KRAS signalling cascades (Tape et al., 2016). KRAS^G12D^ PDAC cell lines grown in co-culture with heterotypic fibroblasts exhibit total proteomes and phospho-proteomes that are distinct from KRAS^G12D^ PDAC cells grown autonomously. The reciprocal signalling between KRAS^G12D^ PDAC cells and fibroblasts involves an IGF1R/AXL-AKT axis that mediates PDAC cell proliferation and apoptosis. The identification of IGF1R/AXL-AKT signalling cascades in reciprocally engaged tumour cells has uncovered additional therapeutic targets acting downstream of oncogenic KRAS^G12D^ and further reinforces the importance of understanding therapy in the context of complex heterocellular TMEs (Tape et al., 2016).

Single cell analyses of both murine and human PDAC have identified 3 distinct CAF cell populations, referred to as myofibroblastic CAFs (myCAFs); inflammatory CAFs (iCAFs); and antigen presenting CAFs (apCAFs) (Sahai et al., 2020). The complex roles attributed to these CAF populations are only just starting to be resolved, however, a link between myCAF phenotypes and tumour promotion is emerging. CAF myofibroblast programmes mediate immunosuppressive TMEs and may be enriched after CRT (Hwang et al., 2020). A limited clinical trial broadly targeting fibroblasts in PDAC was terminated due to disease acceleration (NCT01130142). Further studies are, therefore, required to understand CAF phenotypic heterogeneity and their role in inducing reciprocal signalling cascades in engaged tumour cells before stroma-targeting therapies are a reality in PDAC.

## Future -Omic Approaches for Understanding Intra-Tumoral Heterogeneity and Spatially Resolving PDAC Tumours

The characterisation of PDAC intra-tumoral heterogeneity (ITH) is in its nascent stages. ITH represents an important clinical challenge, as it provides a pool of tumour cell intrinsic variation that may drive cancer progression and lead to the emergence of drug resistance subclones (Gerstung et al., 2020; Dentro et al., 2021). Sub-clonal drug resistance and associated driver mutations are common and often emerge after therapy. Emerging studies in other cancer settings demonstrate that tumour ITH is complex and involves a combination of genomic, transcriptomic, and epigenetic programmes. To fully elucidate the scale and importance of ITH in PDAC integrated multi-omics approaches will be required to deliver a systems level view of tumour evolution and its vulnerabilities.

Profiling chromatin accessibility at single cell resolution using scATAC-seq provides a window into underlying epigenetic regulatory mechanisms that drive cell type differences. scATAC-seq can identify global changes that are not readily apparent at the transcriptome-level and has revealed rare subpopulations of tumour cells that result in therapeutic resistance and/or metastatic progression (Satpathy et al., 2019; Lim et al., 2020; Xu K et al., 2021). The integration of scRNA-seq and scATAC-seq data obtained from the same sample provides highly complementary information about tumour cell transcriptional programmes and the underlying epigenetic regulatory mechanisms that drive them. Recent studies integrating both scRNA-seq and scATAC-seq, have charted cell state changes between precursor lesions and primary disease and have defined complex cellular interactions along a continuum of neoplastic phenotypes (Satpathy et al., 2019). The application of these methodologies to PDAC will likely uncover new phenotypic states and help to determine whether Classical and Basal-like cells exist as a disease continuum. Moreover, complex regulatory information will provide new mechanistic insights into the evolution of PDAC and the selection of drug resistant cell populations.

Beyond spatially resolved transcriptomics new spatial genomic methodologies are emerging. Base Specific *In Situ* Sequencing (BaSISS) uses multiplexed highly sequence specific padlock probes to detect and visualise mutant and wild type alleles in fixed tissue specimens (Lomakin et al., 2021). Multiplexed with existing spatial transcriptomic and imaging modalities BaSiSS can generate large scale quantitative maps of cancer clones harbouring defined mutations. For the first time, specific cancer clones can be imaged and mapped to distinct histological features and associations between specific clones and stromal cell subsets followed over the course of a patient’s treatment journey (Lomakin et al., 2021).

New Mass Spectrometry Imaging (MSI) methods are also now available that allow simultaneous visualisation of drugs, their metabolites, and endogenous compounds within tissue samples. MSI methods such as Matrix-assisted laser desorption ionization (MALDI) or Desorption Electrospray ionisation (DESI) can generate spatially resolved images of specific analytes using fresh frozen tissue sections (Cornett et al., 2007; Inglese et al., 2017; Vaysse et al., 2017). H&E staining, multiplexed immunofluorescence or imaging mass spectrometry can be applied to sequential tissue sections to develop a composite picture of the types of cells, histological features and drug metabolites associated with a defined region of interest. The integration of MSI with complementary imaging modalities will provide important insights into drug pharmacodynamics within complex tissue architectures and highlight distinct tumour regions associated with previously define biomarkers.

## Circulating Biomarkers in PDAC

The rapid identification of circulating biomarkers, obtained from a liquid biopsy, e.g., routine venous phlebotomy, has the potential to facilitate real-time monitoring of a patient’s treatment journey, from early diagnosis to response to therapy. The most extensively used blood-borne biomarker for PDAC is Carbohydrate Antigen 19-9 (CA19-9), which is primarily used to establish a primary diagnosis and to determine recurrence or progression of disease after therapy. CA19-9, however, is limited in its utility as an effective biomarker for PDAC for several reasons. Firstly, it is not specific for PDAC and is elevated in other cancers and benign conditions such as biliary obstructions. Secondly, it has no utility in approximately 10% of Caucasians and 22% of African Americans who are Lewis antigen negative (M. A. Tempero et al., 1987; Poruk et al., 2013; Goonetilleke and Siriwardena 2007).

Circulating blood-borne biomarkers including circulating tumour cells (CTCs), tumour DNA (ctDNA), and extra-cellular vesicles such as exosomes can all be identified in a blood sample. Rapid advancement in the detection and characterisation of these biomarkers, in PDAC and other cancers, point to their potential usefulness in early disease detection and monitoring of therapy response (Crowley et al., 2013; J. Lee et al., 2019; Gall et al., 2019; Alix-Panabières and Pantel 2021; Li et al., 2021).

### Circulating Tumour Cells

Local tissue and lymphatic invasion are common features of early PDAC. The dissemination of cancer cells from the site of primary lesion to surrounding tissues and ultimately distant organs is thought to involve circulating tumour cells (CTCs). CTCs are shed from the primary tumour into the bloodstream and are likely to be enriched for metastatic precursors (Lozar et al., 2019). CTCs are very rare, however, technical advances in methods for CTC isolation now allow for the molecular profiling of CTCs using diverse omic approaches (Yu et al., 2012; Ting et al., 2014; Jiang et al., 2015). The molecular profiling of CTCs, therefore, offers a non-invasive opportunity to identify novel biomarkers for early PDAC detection (J. Lee et al., 2019). In addition, as CTCs represent metastatic precursors molecular profiling may identify drivers of early recurrence and help to develop targeted therapies that inhibit metastatic disease (Gall et al., 2019).

CTCs have been found at all stages of PDAC. Pre-cancerous human pancreatic intraductal papillary mucinous neoplasms (IPMNs) release CTCs in comparable quantities to localised early PDAC (Court et al., 2018). Importantly, several studies have demonstrated that CTCs serve as prognostic biomarkers for PDAC. CTC positivity in the peripheral blood of PDAC patients is significantly correlated with shorter progression free survival and the development of metastases (Bidard et al., 2013; Court et al., 2018). The ability of CTCs to predict therapeutic response is unclear, although pharmacogenomic models of PDAC suggest that CTC profiling can identify patient responders for specific chemotherapy regimens.

Best estimates suggest that CTCs exist at a ratio of one to ten cancer cells per 10 billion normal blood cells in a mL of blood (Miller et al., 2010). CTCs are therefore typically enriched using phenotypic properties, such as size or density, or based on specific cell surface markers (Lianidou et al., 2014; Martini et al., 2019; J.; Lee et al., 2019). Enrichment of CTCs by flow cytometry or microfluidic systems provides sufficient material for omic analysis including WGS (Jiang et al., 2015), RNA-seq (Yu et al., 2012), or single-cell sequencing (Ting et al., 2014). The development of CTC-based biomarkers, therefore, may extend beyond simple CTC enumeration to mutational and expression profiling.

Transcriptomic profiling of CTCs isolated from Genetically Engineered Mouse Models (GEMM) of PDAC identified WNT signalling and aberrant extracellular matrix expression (ECM) as drivers of PDAC metastasis (Yu et al., 2012). Single cell sequencing of CTCs isolated from patients with locally advanced or metastatic pancreatic cancer corroborated findings in murine models showing specific upregulation of the ECM gene SPARC. SPARC expression was highly correlated with the expression of ZEB1 and vimentin, master regulators of Epithelial-to-Mesenchymal transition (EMT) (Lapin et al., 2017).

Franses et al. have recently performed RNA-sequencing of CTCs isolated from the blood of healthy donors, patients with either treatment naïve localised PDAC or metastatic PDAC (Franses et al., 2020). Differential gene expression analysis demonstrated that Mucin (MUC) genes, MUC3A, MUC4, MUC16, and MUC17 genes are significantly enriched in CTCs isolated from treatment naïve localised PDAC and metastatic PDAC versus normal controls. Importantly, correlation analysis identified 3 major subgroups of samples designated as LGALS3-high, WNT5A-high and LIN28B/KLF4-high. These genes are known as “stemness” markers and have been demonstrated to play a role in EMT. Of the “stemness” markers identified, LIN28B alone was found to be prognostic for poor survival. LIN28B plays an important role in modulating stem-like states by binding and blocking the maturation of let-7, a well-characterized tumour suppressor miRNA that targets multiple oncogenes. CRISPR-mediated silencing of LIN28B in cell lines and mouse model systems resulted in less aggressive metastatic phenotypes, primarily through the concomitant upregulation of let-7. CRISPR knock-out of let-7 target HGMA2 or chemical inhibition of LIN28B/let-7 binding mimicked the metastatic phenotypes observed in LIN28B CRISPR models. These results support the development of drugs targeting LIN28B/let-7 phenotypes for the control of metastatic disease.

Future strategies that utilise omic profiling of CTCs will provide important biomarkers and targets for therapeutic development, especially in the identification and control of early metastatic dissemination. The extension of these studies to longitudinal patient cohorts will likely expand our understanding of CTC evolution and identify additional biomarkers associated with therapeutic response. A significant drawback for the use of CTCs as an effective “liquid biopsy” is that they are exceedingly rare in standard blood draws.

### Circulating Tumour DNA

Cell-free tumour DNA comprises a range of extracellular DNA including the fragments of circulating tumour DNA (ctDNAs). ctDNAs are released into the bloodstream by dying tumour cells and can be isolated from patient plasma providing a clinically relevant liquid biopsy analyte for tumour genotyping (Cohen et al., 2018; Jaworski et al., 2020). Somatic mutations and copy number alterations can be detected in ctDNAs by targeted gene sequencing or droplet digital PCR (Sausen et al., 2015; Botrus et al., 2021). These methodologies allow the detection of rare ctDNA mutations with MAFs as low as <0.2% (Le Calvez-Kelm et al., 2016). Whole-genome or whole-exome approaches for ctDNA analysis are complicated by low tumour cell fractions in ctDNA isolates and have not been widely applied in PDAC ctDNA analyses.

The mutational landscape of PDAC is well characterised, with oncogenic mutations in KRAS found in greater than 90% of PDAC (Biankin et al., 2012). The utility of KRAS ctDNA mutations as a prognostic marker has been demonstrated in several studies. Detection of KRAS mutant alleles in PDAC ctDNAs is associated with significantly poorer disease-free and overall survival (Hadano et al., 2016; Tjensvoll et al., 2016; Kruger et al., 2018; Perets et al., 2018; Bernard et al., 2019; Guo et al., 2020). Moreover, KRAS ctDNA mutations are correlated with tumour grade (Le Calvez-Kelm et al., 2016).

The profiling of ctDNAs also serves as a diagnostic tool for early PDAC. CancerSEEK a multi-analyte screening test that can simultaneously detect somatic ctDNA mutations in 16 genes and quantify 8 cancer-associated proteins (carbohydrate antigen 125 (CA-125), CA19-9, CEA, HGF, myeloperoxidase, prolactin, OPN, tissue inhibitor of metalloproteinases 1 (TIMP-1)) can identify cancer in approximately 70% of PDAC patients (Cohen et al., 2018).

Detection of 5-hydroxymethylcytosine (5hmC) in ctDNA may also provide a means for early PDAC diagnosis. Mapping of 5hmC changes in ctDNA between non-cancer and PDAC cohorts identified thousands of genes commonly deregulated in PDAC, including many genes involved in pancreas development (GATA6, GATA4) and pathogenesis (YAP1, PROX1, IGF1). 5hmC sites significantly enriched between PDAC and control groups were sufficient to discriminate PDAC from non-cancer patients (Guler et al., 2020).

Next generation sequencing has consistently found that 25% or more of PDAC tumours harbour actionable mutations (Aguirre et al., 2018; Pishvaian et al., 2020). Similarly, PDAC ctDNAs comprise high-confidence tumour mutations in actionable genes. Botrus et al. recently investigated actionable genetic alterations in ctDNA isolated from the blood of patients with locally advanced and metastatic PDAC. Several therapeutically actionable alterations were identified including mutations in the homologous recombination repair pathway genes BRCA1 (2.1%), BRCA2 (5%), and ATM (7%) (Botrus et al., 2021). The identification of actionable mutations in ctDNAs using a liquid biopsy may significantly improve the time between first detection and therapeutic intervention.

### PDAC Blood Metabolomes

Metabolomics is defined as the quantitative analysis of metabolites (endogenous low molecular weight components <1 kDa) in a biological specimen. Several studies have explored the utility of blood borne metabolites for PDAC early diagnosis, patient stratification and patient monitoring. These studies, while providing some prospects, have failed to identify a single metabolite biomarker that can accurately discriminate PDAC from chronic pancreatitis (CP) (Mayerle et al., 2018).

The development of multi-analyte signatures comprising CA19-9 and several metabolite biomarkers may provide increased specificity for PDAC diagnosis. Mayerle et al., investigated 477 metabolites from patients with CP or PDAC and demonstrated that a panel of nine metabolites (Proline, Sphingomyelin (d18:2,C17:0), Phosphatidylcholine (C18:0,C22:6), Isocitrate, Sphinganine-1-phosphate (d18:0), Histidine, Pyruvate, Ceramide (d18:1,C24:0), Sphingomyelin (d17:1,C18:0)) in conjunction with CA19-9 levels can distinguish PDAC from CP (Mayerle et al., 2018).

The effectiveness of metabolomic profiling for patient stratification has also recently been investigated. Metabolomic profiling of 361 PDAC blood plasma samples identified three subtypes, with sphingolipid metabolism exhibiting differential enrichment between subtypes. Integrative analysis revealed that the 3 identified subtypes do not overlap with previously defined transcriptomic subtyping schemes and are not associated with clinical outcome (Mahajan et al., 2021). While identifying sphingolipid metabolites as potentially important biomarkers in PDAC, the utility of serum based metabolic profiling for patient stratification remains unclear.

### PDAC Exosomes

Exosomes are membrane-bound extracellular vesicles ranging in size between 40 and 160 nm in diameter. Derived from the endosomal compartment, exosomes are secreted from cells and can be readily detected in blood and other bodily fluids (Kalluri 2016; Kalluri and LeBleu 2020). Importantly, exosomes have been demonstrated to contain a multitude of analytes, including nucleic acids (DNA, mRNA, microRNA, long non-coding RNA) (Valadi et al., 2007; Kahlert et al., 2014; Hinger et al., 2018), proteins (Hurwitz et al., 2016; Kowal et al., 2016), lipids (Haraszti et al., 2016), and metabolites (Altadill et al., 2016). The biological role for exosomes as mediators of cell-cell communication now seems established, with exosomes delivering functional cargoes to, or binding membrane bound receptors of recipient cells (Alexander et al., 2015; Hoshino et al., 2015; Muller et al., 2017). The diverse role of exosomes in tumorigenesis, metastasis, immune regulation, and TME remodelling coupled with their long half-life in circulation make exosomes and their cargoes attractive biomarkers for patient diagnosis, prognosis, and biological discovery (Capello et al., 2019; Sun et al., 2020). Moreover, given that exosomes are programmable via genomic and/or proteomic modification exosomes are now being used as therapeutic vehicles (Zitvogel et al., 1998; Kamerkar et al., 2017; Usman et al., 2018; Kugeratski and Kalluri 2021).

Several recent studies suggest that the profiling of exosome cargoes may have clinical utility for PDAC diagnosis. miRNA profiling of exosomes isolated from the plasma of PDAC patients has identified miR-10b, miR-196a, miR451a, miR-21, and miR-17-5p as biomarkers for the diagnosis of early PDAC (Que et al., 2013; Joshi et al., 2015; Y.-F. Xu et al., 2017; Takahasi et al., 2018). In addition, miRNA profiling of exosomes isolated from the plasma of patients with either CP, benign pancreatic tumours (BPTs), or PDAC has demonstrated that specific exosomal miRNAs (miR-21, miR-17-5p) can be used to discriminate PDAC from CP and/or BPTs (Que et al., 2013). Furthermore, analysis of exosomes isolated from the pancreatic juice of PDAC patients or subjects with CP identified exosomal miR-21 and miR-155 as biomarkers capable of differentiating PDAC from CP (Nakamura et al., 2019).

Exosome proteins may also serve as important biomarkers for PDAC diagnosis. Quantitative proteomics has identified core exosomal proteins that are highly expressed in PDAC and other cancer cell types. Glypican-1 (GPC1), a cell surface proteoglycan, is enriched in PDAC-derived exosomes. GPC1 positive exosomes isolated from patient sera were shown to discriminate PDAC patients from healthy volunteers and patients with benign pancreatic disease (Melo et al., 2015). Macrophage migration inhibitory factor (MIF) is also highly expressed in PDAC-derived exosomes. MIF expression is higher in exosomes derived from stage I PDAC patients who later develop liver metastasis (Costa-Silva et al., 2015). Despite the potential of diagnostic specific exosome protein biomarkers in PDAC, future analyses will be required to both validate and extend these findings.

## Knowledge-Guided Platforms for Better Patient Management: Challenges and Opportunities

The early detection of cancer is now a major focus of health care systems around the world. Galleri™, a multi-cancer early detection (MCED) test that can detect over 50 types of cancer from a blood draw has been hailed as a “game changer” for cancer care with the potential to reduce deaths and decrease healthcare costs by detecting cancers early before they metastasise (https://www.galleri.com/). The Galleri™ test, now being trialled by the National Health Service (NHS) in the United Kingdom, employs a methylation-based ctDNA classifier to detect cancer and its location (Braunstein and Ofman 2021; Klein et al., 2021; Nadauld et al., 2021). Galleri™ has been demonstrated to detect Stage I, II and III PDAC with sensitivities ranging from 61.9 to 85.7% (Klein et al., 2021).

The deployment of MCED ctDNA-based tests for the routine early detection of PDAC remain challenging. Firstly, PDAC is rare in the general population and false positive screening may increase costs and result in patient harm from unnecessary procedures (Hruban and Lillemoe 2019; Lennon et al., 2019). Secondly, even though detected at early stages PDAC may still be extremely difficult to treat. For example, several studies demonstrate that venous invasion is a common attribute of PDAC, and as veins within the pancreas empty into the liver, it has been suggested that early liver metastases may be a frequent feature of early stage PDAC (Noe et al., 2018; Hruban et al., 2019; Hong et al., 2020). Beyond early detection, ctDNAs might serve as minimally invasive blood-borne biomarkers for monitoring patient treatment response. Comprehensive longitudinal studies charting ctDNA changes over the course of standardised therapy will be an important first step in making this a reality.

The identification of actionable mutations in an estimated 30–40% of PDAC has amplified calls for the routine genomic profiling of patient tumours. The “Know Your Tumour” trial has performed targeted sequencing on 1856 patient tumours with only 46 patients (2.5%) receiving a selected second line therapy matched to an actionable mutation (Pishvaian et al., 2020). This study points to survival gains for patients who receive a matched therapy based on genomic profiling, however, there is some debate as to whether these gains represent a substantial effect (Pishvaian et al., 2020). A significant difficulty in implementing precision oncology for PDAC is the length of time needed to acquire a patient sample, perform genomic profiling, and guide a patient to a molecularly matched therapy. Precision oncology trials in PDAC have largely focused on advanced disease with genome-matched therapies offered in the second line after standardised chemotherapy. The short survival times of patients with advanced PDAC means that timelines for meaningful intervention are significantly reduced. In addition, the current paradigm of guiding therapy based on single agent matched therapies often results in recurrence as resistant subclones emerge after therapy with repeat biopsies and genomic profiling leading to diminishing returns.

Several other clinical trials, including EPPIC (Enhanced Pancreatic Cancer Profiling for Individualised Care Study; https://www.tfri.ca), Precision Promise (https://www.pancan.org/research/precision-promise/) and ESPAC6, are pursuing multi-omic strategies to better define patient selection for standardised chemotherapy and to accelerate drug development for PDAC. The ESPAC6 clinical trial will assess whether a treatment specific transcriptomic signature can inform patient selection for either gemcitabine or FOLFIRINOX adjuvant therapy. In addition, ESPAC6 will generate a comprehensive cohort of patient-derived organoids. Organotypic models derived from patient samples with matched primary resectable material and clinical response data may represent a more clinically relevant approach for the testing of new drugs and/or biomarker hypotheses. Collectively, these studies will be highly informative and will likely define broader-based multianalyte selection strategies for patient management.

The next phase of omic driven discovery science has arrived. Single cell and single nucleus sequencing are providing new insights into PDAC ITH. Spatial transcriptomic and multiplexed imaging modalities are revealing critical cell-cell interactions, receptor-ligand interactions, and complex tumour architectures that will likely identify a next generation of cancer biomarkers and therapeutic opportunities for PDAC. The utility of single cell transcriptomics is limited for now to discovery; however, single cell data can be obtained from real-world PDAC biopsies (J. J. Lee et al., 2021). Multiplexed imaging modalities, such as MSI, that can generate composite maps of different cell types and drug metabolites may represent a more informative approach to the analysis of clinical samples in the future. Importantly, integrative systems and machine learning approaches are unlocking the potential of these new omic techniques to identify new cancer biomarkers and transition towards next generation knowledge-guided platforms for clinical decision making.
